# Crystal structure of chlorido­(5,10,15,20-tetra­phenyl­porphyrinato-κ^4^ *N*)manganese(III) 2-amino­pyridine disolvate

**DOI:** 10.1107/S205698901500050X

**Published:** 2015-01-17

**Authors:** Wafa Harhouri, Salma Dhifaoui, Shabir Najmudin, Cecilia Bonifácio, Habib Nasri

**Affiliations:** a Laboratoire de Physico-chimie des Matériaux, Faculté des Sciences de Monastir, Avenue de l’Environnement, 5019 Monastir, University of Monastir, Tunisia; b Faculdade de Medicina, Veterinària, Universidade Tecnica de Lisboa, Avenida da Universidade Tecnica, 1300-477 Lisboa, Portugal; c REQUIMTE/CQFB Departamento de Quimica, Faculdade de Ciencias e Tecnologia, Universidade Nova de Lisboa, 2829-516 Caparica, Portugal

**Keywords:** Crystal structure, manganese porphyrin complex, hydrogen bonding

## Abstract

In the title compound, [Mn(C_44_H_28_N_4_)Cl]·2C_5_H_6_N_2_, the Mn^III^ centre is coordinated by four pyrrole N atoms [averaged Mn—N = 2.012 (4) Å] of the tetra­phenyl­porphyrin mol­ecule and one chloride axial ligand [Mn—Cl = 2.4315 (7) Å] in a square-pyramidal geometry. The porphyrin macrocycle exhibits a non-planar conformation with major *ruffling* and *saddling* distortions. In the crystal, two independent solvent mol­ecules form dimers through N—H⋯N hydrogen bonding. In these dimers, one amino N atom has a short Mn⋯N contact of 2.642 (1) Å thus completing the Mn environment in the form of a distorted octa­hedron, and another amino atom generates weak N—H⋯Cl hydrogen bonds, which link further all mol­ecules into chains along the *a* axis.

## Abstract 2

In the title compound, the chlorido­(5,10,15,20-tetra­phenyl­porphyrinato)manganese(III) complex and the hydrogen-bonded dimer of 2-amino­pyridine mol­ecules are linked together by weak N—H⋯Cl hydrogen bonds into chains along the *a* axis.

## Chemical context   

In a continuation of our studies of metalloporphyrins, which are usually used as models of hemoproteins and have various applications in many fields such as catalysis (Amiri *et al.*, 2014 ▸), photodynamic therapy (Kolarova *et al.*, 2005 ▸), conception of sensors (Garg *et al.*, 2013 ▸) or the design of photoluminescent species (Harry *et al.*, 2003 ▸), we report herein the synthesis and crystal structure of the title compound, [Mn(C_44_H_28_N_4_)Cl]·2C_5_H_6_N_2_, (I).
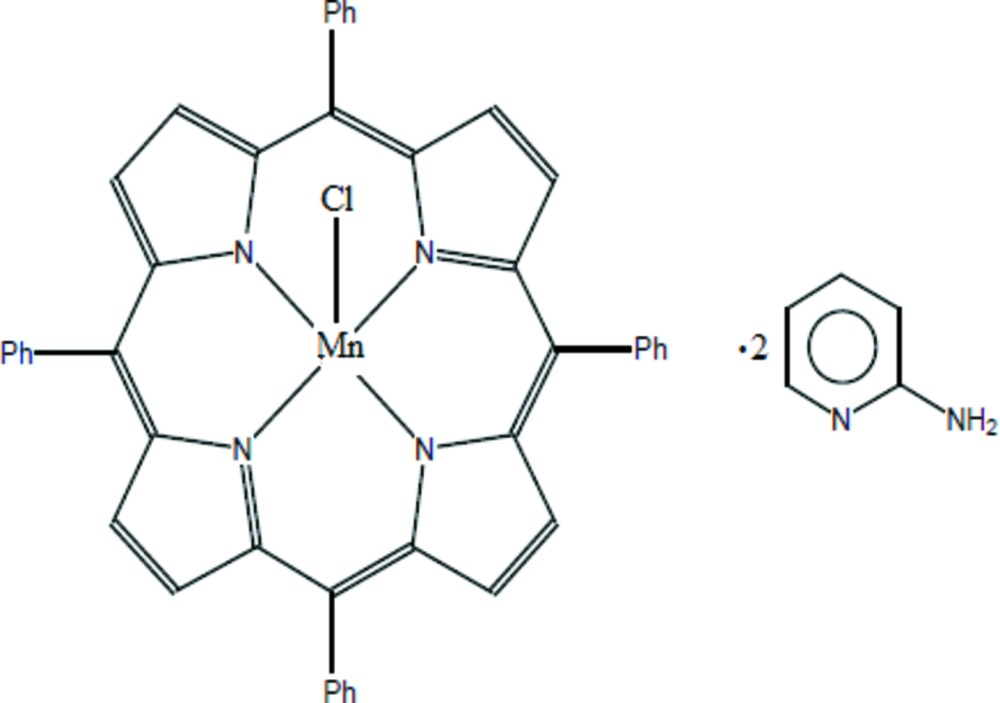


## Structural commentary   

**Figure 1:**
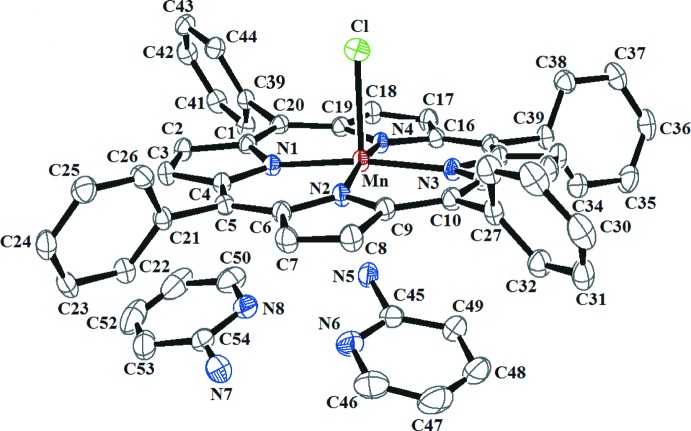
The contents of the asymmetric unit of (I), showing the atomic numbering. Displacement ellipsoids are drawn at the 50% probability level. H atoms are omitted for clarity.

In (I), the central Mn^III^ atom has a square-pyramidal coordination geometry (Fig. 1 ▸). The equatorial plane is formed by four nitro­gen atoms of the porphyrin whereas the apical position is occupied by the chlorido ligand. The asymmetric unit of (I) consists of the [Mn^III^(TPP)Cl] complex (TPP is the 5,10,15,20-tetra­phenyl­porphyrinato ligand) and two 2-amino­pyridine solvent mol­ecules. The average equatorial mangan­ese–N(pyrrole) distance (Mn—N_p_) is 2.012 (4) Å, while the Mn—Cl bond length is 2.4315 (7) Å. The manganese atom is displaced by 0.1616 (5) Å from the 24-atom porphyrin mean plane. The porphyrin core presents a major *ruffling* deformation, as seen in the positions of the *meso* carbons alternatively above and below the mean plane of the 24-atom porphyrin macrocycle, and a *saddle* distortion involving the displacement of the pyrrole rings alternately above and below the porphyrin macrocycle mean plane (Scheidt & Lee, 1987 ▸). This is confirmed by normal structural decomposition (NSD) calculations (Jentzen *et al.*, 1998 ▸), with *ruffling* and *saddle* percentages of 40% and 36%, respectively.

## Supra­molecular features   

**Figure 2:**
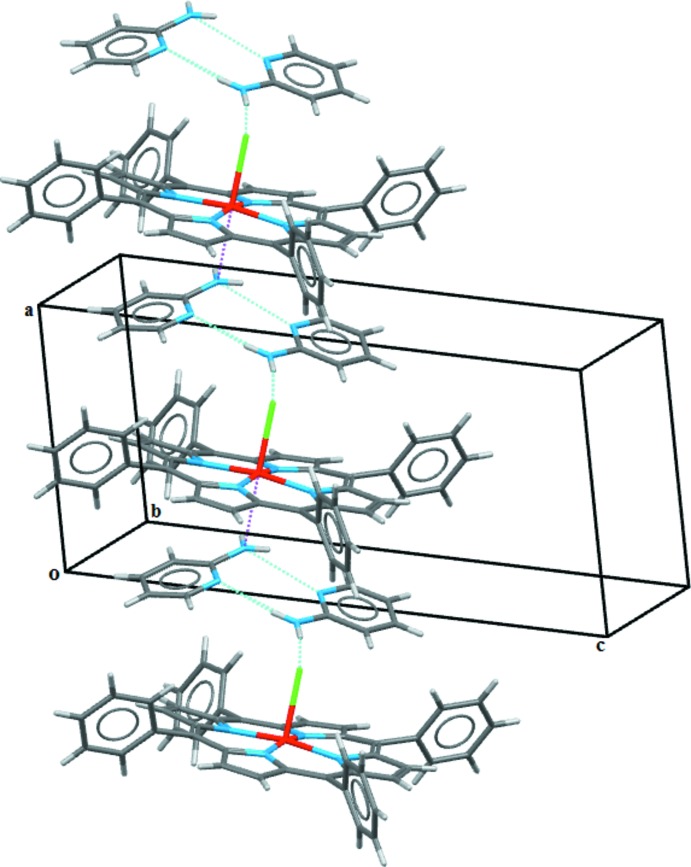
A portion of the crystal packing showing the N—H⋯Cl hydrogen bonds (dotted blue lines) and short Mn⋯N contacts (dashed pink lines).

In the crystal structure, two 2-amino­pyridine solvent mol­ecules are paired into dimers *via* N—H⋯N hydrogen bonds involving the amino groups of these two mol­ecules (Table 1 ▸). In these dimers, one amino atom has a short Mn⋯N contact of 2.642 (1) Å and another amino atom generates weak N—H⋯Cl hydrogen bonds, which further link the components into chains along the *a-*axis direction (Fig. 2 ▸).

## Database survey   

The majority of the known manganese–porphyrin species with halides are penta-coordinated, *e.g.* [Mn^III^(TPP)Cl] (Stute *et al.*, 2013 ▸), [Mn^III^(TPP)Br] and [Mn^III^(TPP)I] (Turner *et al.*, 1998 ▸). Nevertheless, the six-coordinated di­fluoro-mangan­ese(IV) porphyrin species is also known: [Mn^IV^(TMP)F_2_] (TMP is the 5,10,15,20-tetra­mesitylporphyrinato ligand) (Liu *et al.*, 2012 ▸). In the Cambridge Structural Database (CSD, Version 5.35; Groom & Allen, 2014 ▸), there are fourteen chlorido porphyrin structures with a penta-coordinate Mn^III^ atom, five of them with the 5,10,15,20-tetra­phenyl­porphyrin (TPP) ligand. For the known [Mn^III^(Porph)Cl] complexes (Porph = porphyrinato ligand) [CSD refcodes HIFMIS (Cheng & Scheidt, 1996 ▸) and SENMUU (Paulat *et al.*, 2006 ▸)], the equatorial manganese—N(pyrrole) distances (Mn—N_p_) are in the range 2.002 (3)–2.019 (1) Å. This is also the case for (I), where the Mn—N_p_ bond length is 2.012 (4) Å. The Mn—Cl distance of 2.4315 (7) Å in (I) is in agreement with those reported for related compounds [CSD refcodes HIFMIS (Cheng & Scheidt, 1996 ▸) and YEFYAL (Ishikawa *et al.*, 2012 ▸)], with Mn—Cl bond lengths covering the range 2.30–2.66 Å.

## Synthesis and crystallization   

To a solution of [Mn^III^(TPP)Cl] (100 mg, 0.142 mmol) (Cheng & Scheidt, 1996 ▸) in chloro­benzene (10 ml) was added an excess of 2-amino­pyridine **(**50 mg, 0.531 mmol). The reaction mixture was stirred at room temperature for 12 h. Crystals of the title complex were obtained by diffusion of hexa­nes through the chloro­benzene solution.

## Refinement details   

Crystal data, data collection and structure refinement details are summarized in Table 2 ▸. All H atoms were fixed geometrically and treated as riding, with C—H = 0.93, N—H = 0.86 Å and with *U*_iso_(H) = 1.2*U*_eq_(C, N).

## Supplementary Material

Crystal structure: contains datablock(s) I, New_Global_Publ_Block. DOI: 10.1107/S205698901500050X/cv5479sup1.cif

Structure factors: contains datablock(s) I. DOI: 10.1107/S205698901500050X/cv5479Isup2.hkl

CCDC reference: 1042885

Additional supporting information:  crystallographic information; 3D view; checkCIF report

## Figures and Tables

**Table 1:** Hydrogen-bond geometry (, )

*D*H*A*	*D*H	H*A*	*D* *A*	*D*H*A*
N5H5*A*N8	0.86	2.29	2.993(3)	139
N7H7*A*N6	0.86	2.19	3.045(3)	173
N7H7*B*Cl^i^	0.86	2.51	3.358(2)	169

Symmetry code: (i) 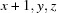.

**Table 2:** Experimental details

Crystal data	
Chemical formula	[Mn(C_44_H_28_N_4_)Cl]2C_5_H_6_N_2_
*M*_r_	891.33
Crystal system, space group	Triclinic, *P* 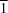
Temperature (K)	180
*a*, *b*, *c* ()	9.9617(4), 12.1247(6), 18.9100(9)
, , ()	92.441(3), 94.699(2), 108.186(2)
*V* (^3^)	2157.01(17)
*Z*	2
Radiation type	Mo *K*
(mm^1^)	0.42
Crystal size (mm)	0.48 0.38 0.16

Data collection	
Diffractometer	Bruker APEXII CCD
Absorption correction	Multi-scan (*SADABS*; Bruker, 2006 ▸)
*T*_min_, *T*_max_	0.701, 0.746
No. of measured, independent and observed [*I* > 2(*I*)] reflections	35821, 8499, 6523
*R*_int_	0.041
(sin /)_max_ (^1^)	0.617

Refinement	
*R*[*F* ^2^ > 2(*F* ^2^)], *wR*(*F* ^2^), *S*	0.041, 0.107, 1.05
No. of reflections	8487
No. of parameters	577
H-atom treatment	H-atom parameters constrained
_max_, _min_ (e ^3^)	0.49, 0.37

Computer programs: *APEX2* and *SAINT* (Bruker, 2006 ▸), *SIR2004* (Burla *et al.*, 2005 ▸), *SHELXL97* (Sheldrick, 2008 ▸, 2015 ▸), *ORTEPIII* (Burnett Johnson, 1996 ▸), *ORTEP-3 for Windows* and *WinGX* (Farrugia, 2012 ▸) and *Mercury* (Macrae *et al.*, 2008 ▸).

## supplementary crystallographic information

### Crystal data

**Table:**

[Mn(C_44_H_28_N_4_)Cl]·2C_5_H_6_N_2_	*Z* = 2
*M**_r_* = 891.33	*F*(000) = 924
Triclinic, *P*1	*D*_x_ = 1.372 Mg m^−^^3^
Hall symbol: -P 1	Mo *K*α radiation, λ = 0.71073 Å
*a* = 9.9617 (4) Å	Cell parameters from 8818 reflections
*b* = 12.1247 (6) Å	θ = 2.2–27.8°
*c* = 18.9100 (9) Å	µ = 0.42 mm^−^^1^
α = 92.441 (3)°	*T* = 180 K
β = 94.699 (2)°	Block, brown
γ = 108.186 (2)°	0.48 × 0.38 × 0.16 mm
*V* = 2157.01 (17) Å^3^	

### Data collection

**Table:**

Bruker APEXII CCD diffractometer	8499 independent reflections
Radiation source: fine-focus sealed tube	6523 reflections with *I* > 2σ(*I*)
Graphite monochromator	*R*_int_ = 0.041
φ and ω scans	θ_max_ = 26.0°, θ_min_ = 1.8°
Absorption correction: multi-scan (*SADABS*; Bruker, 2006)	*h* = −12→12
*T*_min_ = 0.701, *T*_max_ = 0.746	*k* = −14→14
35821 measured reflections	*l* = −20→23

### Refinement

**Table:**

Refinement on *F*^2^	Primary atom site location: structure-invariant direct methods
Least-squares matrix: full	Secondary atom site location: difference Fourier map
*R*[*F*^2^ > 2σ(*F*^2^)] = 0.041	Hydrogen site location: inferred from neighbouring sites
*wR*(*F*^2^) = 0.107	H-atom parameters constrained
*S* = 1.05	*w* = 1/[σ^2^(*F*_o_^2^) + (0.0438*P*)^2^ + 1.0654*P*] where *P* = (*F*_o_^2^ + 2*F*_c_^2^)/3
8487 reflections	(Δ/σ)_max_ = 0.001
577 parameters	Δρ_max_ = 0.49 e Å^−^^3^
0 restraints	Δρ_min_ = −0.37 e Å^−^^3^

### Special details

**Table:**

Geometry. All s.u.'s (except the s.u. in the dihedral angle between two l.s. planes) are estimated using the full covariance matrix. The cell s.u.'s are taken into account individually in the estimation of s.u.'s in distances, angles and torsion angles; correlations between s.u.'s in cell parameters are only used when they are defined by crystal symmetry. An approximate (isotropic) treatment of cell s.u.'s is used for estimating s.u.'s involving l.s. planes.
Refinement. Refinement of F^2^ against ALL reflections. The weighted R-factor wR and goodness of fit S are based on F^2^, conventional R-factors R are based on F, with F set to zero for negative F^2^. The threshold expression of F^2^ > 2sigma(F^2^) is used only for calculating R-factors(gt) etc. and is not relevant to the choice of reflections for refinement. R-factors based on F^2^ are statistically about twice as large as those based on F, and R- factors based on ALL data will be even larger.

### Fractional atomic coordinates and isotropic or equivalent isotropic displacement parameters (Å^2^)

**Table:**

	*x*	*y*	*z*	*U*_iso_*/*U*_eq_	
Mn	0.71850 (3)	0.20739 (3)	0.748853 (16)	0.02309 (10)	
Cl	0.47678 (6)	0.07348 (5)	0.73057 (3)	0.03758 (15)	
N1	0.77802 (17)	0.14667 (15)	0.66018 (9)	0.0234 (4)	
N2	0.80369 (18)	0.10608 (15)	0.80656 (9)	0.0250 (4)	
N3	0.68922 (18)	0.28550 (15)	0.83972 (9)	0.0244 (4)	
N4	0.66655 (18)	0.32903 (15)	0.69325 (9)	0.0234 (4)	
N5	0.9736 (2)	0.36456 (17)	0.76389 (10)	0.0359 (5)	
H5A	0.9784	0.3197	0.7283	0.043*	
H5B	0.9219	0.4093	0.7592	0.043*	
N6	1.1266 (2)	0.29239 (19)	0.83104 (11)	0.0427 (5)	
N7	1.2610 (2)	0.2348 (2)	0.70136 (14)	0.0552 (6)	
H7A	1.2301	0.2540	0.7396	0.066*	
H7B	1.3217	0.1975	0.7035	0.066*	
N8	1.1197 (2)	0.32107 (18)	0.63848 (11)	0.0410 (5)	
C1	0.7439 (2)	0.17043 (18)	0.59150 (11)	0.0233 (4)	
C2	0.7927 (2)	0.10102 (18)	0.54253 (11)	0.0272 (5)	
H2	0.7837	0.1013	0.4932	0.033*	
C3	0.8539 (2)	0.03566 (19)	0.58099 (11)	0.0275 (5)	
H3	0.8953	−0.0173	0.5632	0.033*	
C4	0.8439 (2)	0.06235 (18)	0.65415 (11)	0.0239 (4)	
C5	0.8866 (2)	0.00644 (18)	0.71051 (11)	0.0249 (5)	
C6	0.8707 (2)	0.03070 (18)	0.78147 (11)	0.0257 (5)	
C7	0.9224 (2)	−0.0208 (2)	0.84009 (12)	0.0322 (5)	
H7	0.9723	−0.0739	0.8374	0.039*	
C8	0.8855 (2)	0.0216 (2)	0.89976 (12)	0.0322 (5)	
H8	0.9056	0.0035	0.9459	0.039*	
C9	0.8098 (2)	0.09952 (19)	0.87930 (11)	0.0260 (5)	
C10	0.7539 (2)	0.16021 (19)	0.92676 (11)	0.0270 (5)	
C11	0.6954 (2)	0.24586 (19)	0.90708 (11)	0.0261 (5)	
C12	0.6382 (2)	0.3106 (2)	0.95446 (12)	0.0311 (5)	
H12	0.6278	0.2992	1.0023	0.037*	
C13	0.6022 (2)	0.39102 (19)	0.91726 (11)	0.0301 (5)	
H13	0.5639	0.4462	0.9348	0.036*	
C14	0.6336 (2)	0.37604 (19)	0.84551 (11)	0.0261 (5)	
C15	0.6088 (2)	0.44238 (18)	0.79069 (11)	0.0252 (5)	
C16	0.6228 (2)	0.41858 (18)	0.71991 (11)	0.0245 (5)	
C17	0.5830 (2)	0.47860 (19)	0.66212 (11)	0.0291 (5)	
H17	0.5532	0.5439	0.6660	0.035*	
C18	0.5967 (2)	0.42307 (19)	0.60135 (12)	0.0283 (5)	
H18	0.5755	0.4416	0.5554	0.034*	
C19	0.6499 (2)	0.33038 (18)	0.62017 (11)	0.0241 (4)	
C20	0.6799 (2)	0.25316 (18)	0.57174 (11)	0.0239 (4)	
C21	0.9456 (2)	−0.09079 (19)	0.69477 (11)	0.0255 (5)	
C22	1.0792 (2)	−0.0706 (2)	0.67155 (12)	0.0310 (5)	
H22	1.1331	0.0046	0.6632	0.037*	
C23	1.1328 (2)	−0.1620 (2)	0.66072 (12)	0.0348 (5)	
H23	1.2228	−0.1477	0.6456	0.042*	
C24	1.0536 (3)	−0.2736 (2)	0.67222 (12)	0.0358 (6)	
H24	1.0902	−0.3347	0.6654	0.043*	
C25	0.9196 (3)	−0.2945 (2)	0.69389 (13)	0.0364 (6)	
H25	0.8652	−0.3701	0.7010	0.044*	
C26	0.8656 (2)	−0.2043 (2)	0.70511 (12)	0.0319 (5)	
H26	0.7751	−0.2195	0.7197	0.038*	
C27	0.7585 (2)	0.1328 (2)	1.00308 (11)	0.0297 (5)	
C28	0.6758 (3)	0.0259 (2)	1.02330 (13)	0.0359 (6)	
H28	0.6216	−0.0310	0.9888	0.043*	
C29	0.6734 (3)	0.0032 (2)	1.09463 (14)	0.0430 (6)	
H29	0.6161	−0.0679	1.1077	0.052*	
C30	0.7548 (3)	0.0850 (3)	1.14558 (14)	0.0469 (7)	
H30	0.7520	0.0698	1.1933	0.056*	
C31	0.8410 (3)	0.1896 (3)	1.12667 (13)	0.0432 (7)	
H31	0.8985	0.2441	1.1616	0.052*	
C32	0.8427 (3)	0.2145 (2)	1.05562 (12)	0.0358 (5)	
H32	0.9003	0.2860	1.0432	0.043*	
C33	0.5585 (2)	0.54284 (19)	0.81056 (11)	0.0271 (5)	
C34	0.6532 (3)	0.6437 (2)	0.84414 (13)	0.0379 (6)	
H34	0.7487	0.6501	0.8526	0.045*	
C35	0.6072 (3)	0.7357 (2)	0.86545 (14)	0.0448 (6)	
H35	0.6719	0.8033	0.8880	0.054*	
C36	0.4665 (3)	0.7271 (2)	0.85329 (13)	0.0417 (6)	
H36	0.4357	0.7887	0.8676	0.050*	
C37	0.3715 (3)	0.6272 (2)	0.81989 (14)	0.0418 (6)	
H37	0.2760	0.6211	0.8118	0.050*	
C38	0.4171 (2)	0.5354 (2)	0.79812 (13)	0.0347 (5)	
H38	0.3521	0.4684	0.7750	0.042*	
C39	0.6393 (2)	0.25768 (18)	0.49454 (11)	0.0243 (4)	
C40	0.7006 (2)	0.35470 (19)	0.45743 (11)	0.0289 (5)	
H40	0.7709	0.4185	0.4807	0.035*	
C41	0.6578 (2)	0.3570 (2)	0.38642 (12)	0.0322 (5)	
H41	0.6983	0.4227	0.3624	0.039*	
C42	0.5555 (2)	0.2625 (2)	0.35099 (12)	0.0348 (5)	
H42	0.5275	0.2643	0.3030	0.042*	
C43	0.4945 (2)	0.1651 (2)	0.38668 (12)	0.0343 (5)	
H43	0.4262	0.1008	0.3627	0.041*	
C44	0.5351 (2)	0.16335 (19)	0.45802 (11)	0.0290 (5)	
H44	0.4922	0.0982	0.4821	0.035*	
C45	1.0496 (2)	0.3647 (2)	0.82897 (13)	0.0367 (6)	
C46	1.1971 (3)	0.2868 (3)	0.89372 (17)	0.0570 (8)	
H46	1.2503	0.2360	0.8960	0.068*	
C47	1.1952 (3)	0.3518 (3)	0.95452 (16)	0.0635 (9)	
H47	1.2454	0.3454	0.9970	0.076*	
C48	1.1166 (3)	0.4267 (3)	0.95052 (16)	0.0600 (8)	
H48	1.1145	0.4732	0.9905	0.072*	
C49	1.0412 (3)	0.4330 (2)	0.88792 (14)	0.0474 (7)	
H49	0.9857	0.4820	0.8850	0.057*	
C50	1.0728 (3)	0.3510 (2)	0.57616 (15)	0.0513 (7)	
H50	1.0070	0.3910	0.5762	0.062*	
C51	1.1165 (4)	0.3258 (3)	0.51199 (16)	0.0669 (10)	
H51	1.0816	0.3484	0.4698	0.080*	
C52	1.2135 (4)	0.2663 (3)	0.51215 (19)	0.0770 (12)	
H52	1.2454	0.2483	0.4696	0.092*	
C53	1.2625 (3)	0.2338 (3)	0.57395 (19)	0.0633 (9)	
H53	1.3272	0.1927	0.5743	0.076*	
C54	1.2141 (3)	0.2631 (2)	0.63784 (15)	0.0436 (6)	

### Atomic displacement parameters (Å^2^)

**Table:**

	*U*^11^	*U*^22^	*U*^33^	*U*^12^	*U*^13^	*U*^23^
Mn	0.02838 (18)	0.02739 (19)	0.01763 (17)	0.01494 (14)	0.00212 (13)	0.00061 (13)
Cl	0.0315 (3)	0.0396 (3)	0.0404 (4)	0.0096 (2)	0.0044 (2)	0.0016 (3)
N1	0.0267 (9)	0.0253 (9)	0.0205 (9)	0.0118 (7)	0.0020 (7)	0.0010 (7)
N2	0.0288 (9)	0.0303 (10)	0.0199 (9)	0.0154 (8)	0.0026 (7)	0.0002 (8)
N3	0.0285 (9)	0.0295 (10)	0.0189 (9)	0.0147 (8)	0.0014 (7)	0.0009 (7)
N4	0.0273 (9)	0.0267 (10)	0.0186 (9)	0.0124 (7)	0.0021 (7)	−0.0004 (7)
N5	0.0387 (11)	0.0423 (12)	0.0300 (11)	0.0197 (9)	−0.0001 (9)	−0.0066 (9)
N6	0.0391 (12)	0.0477 (13)	0.0399 (13)	0.0146 (10)	−0.0048 (10)	−0.0007 (10)
N7	0.0464 (13)	0.0565 (15)	0.0671 (18)	0.0259 (12)	0.0012 (12)	−0.0122 (13)
N8	0.0362 (11)	0.0405 (12)	0.0412 (13)	0.0052 (9)	0.0054 (10)	−0.0041 (10)
C1	0.0255 (10)	0.0250 (11)	0.0191 (11)	0.0075 (9)	0.0025 (8)	0.0010 (9)
C2	0.0345 (12)	0.0296 (12)	0.0192 (11)	0.0128 (10)	0.0039 (9)	−0.0003 (9)
C3	0.0326 (11)	0.0284 (12)	0.0251 (12)	0.0141 (9)	0.0072 (9)	−0.0008 (9)
C4	0.0268 (11)	0.0246 (11)	0.0219 (11)	0.0106 (9)	0.0033 (9)	0.0001 (9)
C5	0.0242 (10)	0.0267 (12)	0.0251 (12)	0.0103 (9)	0.0020 (9)	−0.0012 (9)
C6	0.0271 (11)	0.0291 (12)	0.0243 (12)	0.0147 (9)	0.0004 (9)	0.0004 (9)
C7	0.0367 (12)	0.0390 (14)	0.0286 (13)	0.0236 (11)	0.0005 (10)	0.0031 (10)
C8	0.0391 (13)	0.0416 (14)	0.0220 (12)	0.0223 (11)	−0.0003 (10)	0.0041 (10)
C9	0.0292 (11)	0.0319 (12)	0.0196 (11)	0.0141 (9)	−0.0002 (9)	0.0020 (9)
C10	0.0290 (11)	0.0347 (13)	0.0196 (11)	0.0139 (10)	0.0008 (9)	0.0016 (9)
C11	0.0290 (11)	0.0331 (12)	0.0186 (11)	0.0138 (9)	0.0011 (9)	−0.0004 (9)
C12	0.0394 (13)	0.0406 (14)	0.0186 (11)	0.0202 (11)	0.0042 (9)	0.0008 (10)
C13	0.0379 (12)	0.0336 (13)	0.0237 (12)	0.0191 (10)	0.0038 (10)	−0.0026 (10)
C14	0.0284 (11)	0.0292 (12)	0.0237 (11)	0.0140 (9)	0.0018 (9)	−0.0014 (9)
C15	0.0260 (11)	0.0263 (11)	0.0252 (11)	0.0113 (9)	0.0027 (9)	−0.0002 (9)
C16	0.0254 (10)	0.0253 (11)	0.0247 (12)	0.0106 (9)	0.0032 (9)	0.0024 (9)
C17	0.0366 (12)	0.0290 (12)	0.0273 (12)	0.0183 (10)	0.0033 (10)	0.0035 (10)
C18	0.0349 (12)	0.0323 (12)	0.0224 (12)	0.0175 (10)	0.0006 (9)	0.0042 (9)
C19	0.0254 (10)	0.0270 (11)	0.0212 (11)	0.0102 (9)	0.0018 (8)	0.0018 (9)
C20	0.0253 (10)	0.0256 (11)	0.0198 (11)	0.0070 (9)	0.0017 (8)	0.0020 (9)
C21	0.0302 (11)	0.0321 (12)	0.0185 (11)	0.0170 (10)	0.0003 (9)	−0.0003 (9)
C22	0.0341 (12)	0.0315 (13)	0.0307 (13)	0.0145 (10)	0.0063 (10)	0.0020 (10)
C23	0.0337 (12)	0.0458 (15)	0.0326 (13)	0.0224 (11)	0.0097 (10)	0.0015 (11)
C24	0.0459 (14)	0.0394 (14)	0.0302 (13)	0.0261 (12)	0.0021 (11)	−0.0037 (11)
C25	0.0400 (13)	0.0284 (13)	0.0417 (15)	0.0131 (11)	0.0010 (11)	0.0008 (11)
C26	0.0287 (11)	0.0351 (13)	0.0342 (13)	0.0133 (10)	0.0041 (10)	0.0022 (10)
C27	0.0370 (12)	0.0413 (14)	0.0201 (11)	0.0252 (11)	0.0042 (9)	0.0031 (10)
C28	0.0417 (13)	0.0431 (15)	0.0294 (13)	0.0216 (11)	0.0052 (10)	0.0065 (11)
C29	0.0489 (15)	0.0558 (17)	0.0366 (15)	0.0303 (13)	0.0126 (12)	0.0187 (13)
C30	0.0550 (16)	0.077 (2)	0.0263 (14)	0.0421 (16)	0.0109 (12)	0.0179 (14)
C31	0.0506 (15)	0.0693 (19)	0.0228 (13)	0.0410 (15)	−0.0052 (11)	−0.0058 (12)
C32	0.0411 (13)	0.0448 (15)	0.0267 (13)	0.0223 (11)	−0.0002 (10)	0.0002 (11)
C33	0.0373 (12)	0.0295 (12)	0.0202 (11)	0.0173 (10)	0.0078 (9)	0.0036 (9)
C34	0.0382 (13)	0.0354 (14)	0.0416 (15)	0.0149 (11)	0.0042 (11)	−0.0057 (11)
C35	0.0573 (17)	0.0303 (14)	0.0462 (16)	0.0141 (12)	0.0080 (13)	−0.0096 (11)
C36	0.0632 (17)	0.0386 (15)	0.0366 (15)	0.0320 (13)	0.0175 (13)	0.0041 (12)
C37	0.0437 (14)	0.0479 (16)	0.0450 (16)	0.0278 (13)	0.0136 (12)	0.0066 (13)
C38	0.0353 (12)	0.0339 (13)	0.0377 (14)	0.0145 (10)	0.0059 (10)	0.0014 (11)
C39	0.0282 (11)	0.0279 (12)	0.0206 (11)	0.0143 (9)	0.0023 (9)	0.0009 (9)
C40	0.0327 (12)	0.0291 (12)	0.0259 (12)	0.0113 (10)	0.0026 (9)	0.0004 (9)
C41	0.0390 (13)	0.0362 (13)	0.0274 (13)	0.0185 (11)	0.0083 (10)	0.0092 (10)
C42	0.0403 (13)	0.0513 (16)	0.0199 (12)	0.0248 (12)	0.0012 (10)	0.0044 (11)
C43	0.0339 (12)	0.0397 (14)	0.0282 (13)	0.0132 (11)	−0.0038 (10)	−0.0074 (10)
C44	0.0327 (12)	0.0296 (12)	0.0252 (12)	0.0107 (10)	0.0028 (9)	0.0026 (9)
C45	0.0294 (12)	0.0419 (14)	0.0351 (14)	0.0068 (11)	0.0026 (10)	−0.0006 (11)
C46	0.0472 (16)	0.062 (2)	0.060 (2)	0.0181 (14)	−0.0072 (14)	0.0082 (16)
C47	0.0538 (18)	0.088 (2)	0.0372 (17)	0.0111 (17)	−0.0118 (14)	0.0014 (16)
C48	0.0478 (17)	0.078 (2)	0.0399 (17)	0.0036 (16)	0.0007 (13)	−0.0175 (15)
C49	0.0413 (15)	0.0527 (17)	0.0417 (16)	0.0079 (13)	0.0027 (12)	−0.0129 (13)
C50	0.0511 (16)	0.0459 (17)	0.0456 (17)	−0.0001 (13)	0.0015 (13)	0.0027 (13)
C51	0.081 (2)	0.055 (2)	0.0391 (18)	−0.0149 (18)	0.0059 (16)	−0.0031 (14)
C52	0.085 (3)	0.062 (2)	0.057 (2)	−0.0197 (19)	0.037 (2)	−0.0230 (18)
C53	0.0516 (17)	0.0527 (19)	0.076 (2)	0.0002 (14)	0.0282 (17)	−0.0235 (17)
C54	0.0337 (13)	0.0394 (15)	0.0496 (17)	0.0016 (11)	0.0061 (12)	−0.0129 (12)

### Geometric parameters (Å, º)

**Table:**

Mn—N2	2.0083 (17)	C22—H22	0.9300
Mn—N1	2.0089 (16)	C23—C24	1.374 (3)
Mn—N3	2.0127 (17)	C23—H23	0.9300
Mn—N4	2.0169 (16)	C24—C25	1.379 (3)
Mn—Cl	2.4351 (7)	C24—H24	0.9300
N1—C1	1.381 (3)	C25—C26	1.376 (3)
N1—C4	1.382 (2)	C25—H25	0.9300
N2—C9	1.378 (3)	C26—H26	0.9300
N2—C6	1.379 (3)	C27—C32	1.390 (3)
N3—C14	1.380 (3)	C27—C28	1.391 (3)
N3—C11	1.384 (3)	C28—C29	1.389 (3)
N4—C19	1.380 (3)	C28—H28	0.9300
N4—C16	1.381 (3)	C29—C30	1.363 (4)
N5—C45	1.391 (3)	C29—H29	0.9300
N5—H5A	0.8600	C30—C31	1.372 (4)
N5—H5B	0.8600	C30—H30	0.9300
N6—C45	1.333 (3)	C31—C32	1.390 (3)
N6—C46	1.343 (3)	C31—H31	0.9300
N7—C54	1.350 (3)	C32—H32	0.9300
N7—H7A	0.8600	C33—C34	1.380 (3)
N7—H7B	0.8600	C33—C38	1.383 (3)
N8—C54	1.338 (3)	C34—C35	1.388 (3)
N8—C50	1.338 (3)	C34—H34	0.9300
C1—C20	1.394 (3)	C35—C36	1.372 (4)
C1—C2	1.438 (3)	C35—H35	0.9300
C2—C3	1.344 (3)	C36—C37	1.372 (4)
C2—H2	0.9300	C36—H36	0.9300
C3—C4	1.426 (3)	C37—C38	1.386 (3)
C3—H3	0.9300	C37—H37	0.9300
C4—C5	1.394 (3)	C38—H38	0.9300
C5—C6	1.394 (3)	C39—C44	1.392 (3)
C5—C21	1.502 (3)	C39—C40	1.393 (3)
C6—C7	1.432 (3)	C40—C41	1.379 (3)
C7—C8	1.345 (3)	C40—H40	0.9300
C7—H7	0.9300	C41—C42	1.376 (3)
C8—C9	1.428 (3)	C41—H41	0.9300
C8—H8	0.9300	C42—C43	1.381 (3)
C9—C10	1.392 (3)	C42—H42	0.9300
C10—C11	1.390 (3)	C43—C44	1.379 (3)
C10—C27	1.495 (3)	C43—H43	0.9300
C11—C12	1.431 (3)	C44—H44	0.9300
C12—C13	1.345 (3)	C45—C49	1.384 (3)
C12—H12	0.9300	C46—C47	1.371 (4)
C13—C14	1.434 (3)	C46—H46	0.9300
C13—H13	0.9300	C47—C48	1.372 (4)
C14—C15	1.391 (3)	C47—H47	0.9300
C15—C16	1.385 (3)	C48—C49	1.367 (4)
C15—C33	1.500 (3)	C48—H48	0.9300
C16—C17	1.435 (3)	C49—H49	0.9300
C17—C18	1.345 (3)	C50—C51	1.376 (4)
C17—H17	0.9300	C50—H50	0.9300
C18—C19	1.430 (3)	C51—C52	1.374 (5)
C18—H18	0.9300	C51—H51	0.9300
C19—C20	1.397 (3)	C52—C53	1.351 (5)
C20—C39	1.490 (3)	C52—H52	0.9300
C21—C22	1.387 (3)	C53—C54	1.409 (4)
C21—C26	1.390 (3)	C53—H53	0.9300
C22—C23	1.386 (3)		
			
N2—Mn—N1	89.86 (7)	C24—C23—C22	120.4 (2)
N2—Mn—N3	89.23 (7)	C24—C23—H23	119.8
N1—Mn—N3	171.24 (7)	C22—C23—H23	119.8
N2—Mn—N4	170.21 (7)	C23—C24—C25	119.5 (2)
N1—Mn—N4	89.56 (7)	C23—C24—H24	120.2
N3—Mn—N4	89.85 (7)	C25—C24—H24	120.2
N2—Mn—Cl	97.32 (5)	C26—C25—C24	120.6 (2)
N1—Mn—Cl	94.06 (5)	C26—C25—H25	119.7
N3—Mn—Cl	94.70 (5)	C24—C25—H25	119.7
N4—Mn—Cl	92.47 (5)	C25—C26—C21	120.4 (2)
C1—N1—C4	106.00 (16)	C25—C26—H26	119.8
C1—N1—Mn	125.95 (13)	C21—C26—H26	119.8
C4—N1—Mn	127.55 (13)	C32—C27—C28	118.5 (2)
C9—N2—C6	106.07 (16)	C32—C27—C10	120.9 (2)
C9—N2—Mn	127.08 (13)	C28—C27—C10	120.6 (2)
C6—N2—Mn	126.83 (14)	C29—C28—C27	120.6 (2)
C14—N3—C11	106.01 (16)	C29—C28—H28	119.7
C14—N3—Mn	126.43 (14)	C27—C28—H28	119.7
C11—N3—Mn	126.57 (14)	C30—C29—C28	120.2 (3)
C19—N4—C16	106.07 (16)	C30—C29—H29	119.9
C19—N4—Mn	126.53 (13)	C28—C29—H29	119.9
C16—N4—Mn	126.92 (13)	C29—C30—C31	120.2 (2)
C45—N5—H5A	120.0	C29—C30—H30	119.9
C45—N5—H5B	120.0	C31—C30—H30	119.9
H5A—N5—H5B	120.0	C30—C31—C32	120.3 (3)
C45—N6—C46	117.3 (2)	C30—C31—H31	119.8
C54—N7—H7A	120.0	C32—C31—H31	119.8
C54—N7—H7B	120.0	C31—C32—C27	120.2 (2)
H7A—N7—H7B	120.0	C31—C32—H32	119.9
C54—N8—C50	117.7 (2)	C27—C32—H32	119.9
N1—C1—C20	126.20 (18)	C34—C33—C38	118.8 (2)
N1—C1—C2	109.18 (17)	C34—C33—C15	119.9 (2)
C20—C1—C2	124.51 (19)	C38—C33—C15	121.3 (2)
C3—C2—C1	107.55 (19)	C33—C34—C35	120.6 (2)
C3—C2—H2	126.2	C33—C34—H34	119.7
C1—C2—H2	126.2	C35—C34—H34	119.7
C2—C3—C4	107.50 (18)	C36—C35—C34	120.2 (3)
C2—C3—H3	126.3	C36—C35—H35	119.9
C4—C3—H3	126.3	C34—C35—H35	119.9
N1—C4—C5	125.35 (18)	C37—C36—C35	119.7 (2)
N1—C4—C3	109.74 (17)	C37—C36—H36	120.1
C5—C4—C3	124.80 (19)	C35—C36—H36	120.1
C6—C5—C4	123.90 (19)	C36—C37—C38	120.3 (2)
C6—C5—C21	116.96 (18)	C36—C37—H37	119.8
C4—C5—C21	119.04 (18)	C38—C37—H37	119.8
N2—C6—C5	126.34 (19)	C33—C38—C37	120.4 (2)
N2—C6—C7	109.38 (18)	C33—C38—H38	119.8
C5—C6—C7	124.28 (19)	C37—C38—H38	119.8
C8—C7—C6	107.47 (19)	C44—C39—C40	118.3 (2)
C8—C7—H7	126.3	C44—C39—C20	119.52 (18)
C6—C7—H7	126.3	C40—C39—C20	122.15 (19)
C7—C8—C9	107.47 (19)	C41—C40—C39	120.6 (2)
C7—C8—H8	126.3	C41—C40—H40	119.7
C9—C8—H8	126.3	C39—C40—H40	119.7
N2—C9—C10	126.17 (19)	C42—C41—C40	120.3 (2)
N2—C9—C8	109.58 (18)	C42—C41—H41	119.8
C10—C9—C8	124.24 (19)	C40—C41—H41	119.8
C11—C10—C9	123.46 (19)	C41—C42—C43	120.0 (2)
C11—C10—C27	118.20 (18)	C41—C42—H42	120.0
C9—C10—C27	118.34 (18)	C43—C42—H42	120.0
N3—C11—C10	125.52 (19)	C44—C43—C42	119.8 (2)
N3—C11—C12	109.44 (18)	C44—C43—H43	120.1
C10—C11—C12	124.97 (19)	C42—C43—H43	120.1
C13—C12—C11	107.56 (19)	C43—C44—C39	120.9 (2)
C13—C12—H12	126.2	C43—C44—H44	119.5
C11—C12—H12	126.2	C39—C44—H44	119.5
C12—C13—C14	107.46 (19)	N6—C45—C49	122.7 (2)
C12—C13—H13	126.3	N6—C45—N5	115.6 (2)
C14—C13—H13	126.3	C49—C45—N5	121.7 (2)
N3—C14—C15	126.07 (18)	N6—C46—C47	123.7 (3)
N3—C14—C13	109.49 (18)	N6—C46—H46	118.1
C15—C14—C13	124.43 (19)	C47—C46—H46	118.1
C16—C15—C14	124.14 (19)	C46—C47—C48	117.7 (3)
C16—C15—C33	119.05 (18)	C46—C47—H47	121.2
C14—C15—C33	116.77 (18)	C48—C47—H47	121.2
N4—C16—C15	125.90 (18)	C49—C48—C47	120.2 (3)
N4—C16—C17	109.38 (18)	C49—C48—H48	119.9
C15—C16—C17	124.55 (19)	C47—C48—H48	119.9
C18—C17—C16	107.37 (19)	C48—C49—C45	118.4 (3)
C18—C17—H17	126.3	C48—C49—H49	120.8
C16—C17—H17	126.3	C45—C49—H49	120.8
C17—C18—C19	107.57 (19)	N8—C50—C51	123.7 (3)
C17—C18—H18	126.2	N8—C50—H50	118.2
C19—C18—H18	126.2	C51—C50—H50	118.2
N4—C19—C20	125.45 (18)	C52—C51—C50	117.9 (3)
N4—C19—C18	109.55 (18)	C52—C51—H51	121.0
C20—C19—C18	125.00 (19)	C50—C51—H51	121.0
C1—C20—C19	123.73 (19)	C53—C52—C51	120.1 (3)
C1—C20—C39	117.83 (18)	C53—C52—H52	119.9
C19—C20—C39	118.43 (18)	C51—C52—H52	119.9
C22—C21—C26	118.8 (2)	C52—C53—C54	119.1 (3)
C22—C21—C5	122.0 (2)	C52—C53—H53	120.5
C26—C21—C5	119.19 (18)	C54—C53—H53	120.5
C23—C22—C21	120.3 (2)	N8—C54—N7	116.7 (2)
C23—C22—H22	119.8	N8—C54—C53	121.5 (3)
C21—C22—H22	119.8	N7—C54—C53	121.8 (3)

### Hydrogen-bond geometry (Å, º)

**Table:**

*D*—H···*A*	*D*—H	H···*A*	*D*···*A*	*D*—H···*A*
N5—H5*A*···N8	0.86	2.29	2.993 (3)	139
N7—H7*A*···N6	0.86	2.19	3.045 (3)	173
N7—H7*B*···Cl^i^	0.86	2.51	3.358 (2)	169

Symmetry code: (i) *x*+1, *y*, *z*.
